# A Fine-Grained and Privacy-Preserving Query Scheme for Fog Computing-Enhanced Location-Based Service

**DOI:** 10.3390/s17071611

**Published:** 2017-07-11

**Authors:** Xue Yang, Fan Yin, Xiaohu Tang

**Affiliations:** The Information Security and National Computing Grid Laboratory, Southwest Jiaotong University, Chengdu 610031, China; yinfan519@gmail.com (F.Y.); xhutang@swjtu.edu.cn (X.T.)

**Keywords:** location-based services (LBS), fog computing, low-latency, fine-grained, privacy-preserving

## Abstract

Location-based services (LBS), as one of the most popular location-awareness applications, has been further developed to achieve low-latency with the assistance of fog computing. However, privacy issues remain a research challenge in the context of fog computing. Therefore, in this paper, we present a fine-grained and privacy-preserving query scheme for fog computing-enhanced location-based services, hereafter referred to as FGPQ. In particular, mobile users can obtain the fine-grained searching result satisfying not only the given spatial range but also the searching content. Detailed privacy analysis shows that our proposed scheme indeed achieves the privacy preservation for the LBS provider and mobile users. In addition, extensive performance analyses and experiments demonstrate that the FGPQ scheme can significantly reduce computational and communication overheads and ensure the low-latency, which outperforms existing state-of-the art schemes. Hence, our proposed scheme is more suitable for real-time LBS searching.

## 1. Introduction

With the rapid development of the Internet of Things (IoTs), fog computing has been presented by Cisco in 2012 [1] as a supplement to cloud computing. Based on its network edge computing feature, fog computing is able to provide location-awareness applications, where the location-based service (LBS) is the most popular [2,3]. With the assistance of fog computing, the LBS providers can outsource the LBS data to fog nodes and thus provide people a convenient life [4]. For instance, through the LBS provided by a local fog node, a mobile user can query and search points of interest (POIs) within a given distance to his/her location [5,6]. Actually, LBS query has become one of the killer applications in LBS, which allows mobile users to query a LBS provider (such as Google or Bing maps) to obtain the detailed information about POIs in their vicinity (e.g., restaurants, hospitals, etc.). For example, when a tourist arrives at a new place, it is very necessary to query some interesting places, such as hotels, restaurants, scenic spots and so on, based on the LBS. Hence, we focus on the LBS query in this paper.

Although LBS can benefit people a lot, the submitted queries may lead to some sensitive information about users being revealed, such as hobbies, current location and the location where mobile users will reach [7,8]. To clearly illustrate the privacy challenge in LBS query, we consider the following scenario, where a mobile user wants to find a hospital by exposing his location to a local fog node. Thus, this fog node can infer user’s location where he will reach according to his request (the content of the matched POIs). However, even worse, the health condition that is very sensitive to this user, may be disclosed. Conceivably, without the privacy assurance in fog computing, the LBS can not continue to its flourish because users’ personal trajectory and hobby may be exposed. In addition, owing to the mobility of mobile users, the low-latency is very critical for the LBS query [9]. For example, when a driver wants to find the nearest restaurant on a heavy-traffic road, he has to keep driving while waiting for the response to ensure smooth traffic. If the response is slow, the result may already not satisfy the current spatial range of this diver, i.e., the driver has traveled far away. Therefore, latency should also be taken into account.

Recently, many privacy-preserving LBS schemes have been proposed to prevent the location privacy of LBS users. For examples, location obfuscation technology has been presented to protect users’ precise locations from disclosing [10,11]. In addition, spatial cloaking technology has been proposed to conceal the precise location in a cloaking area [12,13]. However, the accuracy of the matched results in these schemes are not satisfactory, since the locations submitted by users are not precise. In order to ensure the data privacy and improve the accuracy of results simultaneously, privacy information retrieval (PIR) [14] has been applied to the LBS query [15,16]. Unfortunately, as mentioned in [17], PIR needs to transmit the entire LBS data to the mobile users, which brings about the heavy communication burden, especially for big data. In addition, a mobile user has to additionally access the LBS database’s index data in a privacy-preserving way, which leads to the linear calculations of all LBS data. Hence, the communication and computational costs are tremendous, which is not cost-efficient for mobile users. To overcome this disadvantage, some efficient cryptographic algorithm-based schemes have been developed for the LBS query, where most of these schemes focused on cloud computing, as it can offer on-demand and scalable storage and unlimited computing capacity, especially for big data processing. For examples, Li et al. [18] discussed an efficient privacy-preserving location-based query over outsourced encrypted data by coding and bilinear pairing techniques; Zhu et al. [19] proposed a new efficient and privacy-preserving LBS query scheme in the outsourced cloud; Peng et al. [20] developed the collaborative trajectory privacy-preserving scheme for the trajectory privacy of continuous queries. However, with vast LBS requests generated everyday, the transmission of the extraordinarily huge volume data to the cloud has resulted in unbearable transmission latency, which will degrade the quality of service to end users, especially mobile users [9,21,22]. As a result, privacy-preserving LBS query schemes designed for cloud computing may not satisfy the low-latency.

Hence, the content of fog computing has been introduced to make up the disadvantage of the centralized processing in the cloud [1]. As far as we know, existing schemes in fog computing mainly concentrated on location privacy in wireless sensor networks [23] or real-time navigation of vehicular ad hoc networks [24]. Nevertheless, few fog computing-based schemes considered the privacy issue in the LBS query. Moreover, almost all state-of-the-art LBS query schemes only take the location matching into consideration without considering content matching, and thus this is not fine-grained. For instance, when a mobile user wants to query a hospital within 500 m, the returned results in existing schemes are all the LBS data meeting the spatial range (within 500 m). That is, in addition to the hospital, the result may include a restaurant, a park, or a mall and so on. In fact, these results are not necessary for LBS users, and it will lead to additional communication and computational costs, e.g., users have to decrypt all the returned ciphertexts to find the point of interest.

To not only ensure the privacy-preserving and low-latency, but also achieve the fine-grained query result, in this paper, we propose a fine-grained and privacy-preserving query scheme for fog computing-enhanced location-based service (FGPQ). The contributions of this paper are threefold as follows.

- First, we present our FGPQ scheme, which is characterized by employing the bilinear pairing [25] and the asymmetric scalar-product preserving encryption (ASPE) [26] to realize the LBS searching. In addition to satisfying the given spatial range, the searching result satisfies the given searching content, which is not considered in many up-to-date schemes.
- Secondly, we give detailed privacy analysis to show that our proposed FGPQ indeed achieves the privacy preservation of both the LBS provider and mobile users.
- Finally, we theoretically analyze the computational and communication overheads, and run extensive experiments to demonstrate that our FGPQ scheme is more efficient than the EPLQ scheme [18] and EPQ scheme [19]. In addition, the latency analysis shows that our FGPQ scheme is really low-latency, which is suitable for the real-time LBS query.

The remainder of this paper is organized as follows. In Section 2, we introduce our system model and design goals. Then, we describe some preliminaries in Section 3. In Section 4, we present our FGPQ scheme, followed by privacy analysis and performance analysis in Section 5 and Section 6, respectively. Finally, we draw our conclusions in Section 7.

## 2. System Model and Design Goals

In this section, we formalize the system model used in this paper, and identify design goals.

### 2.1. System Model

**Figure 1:**
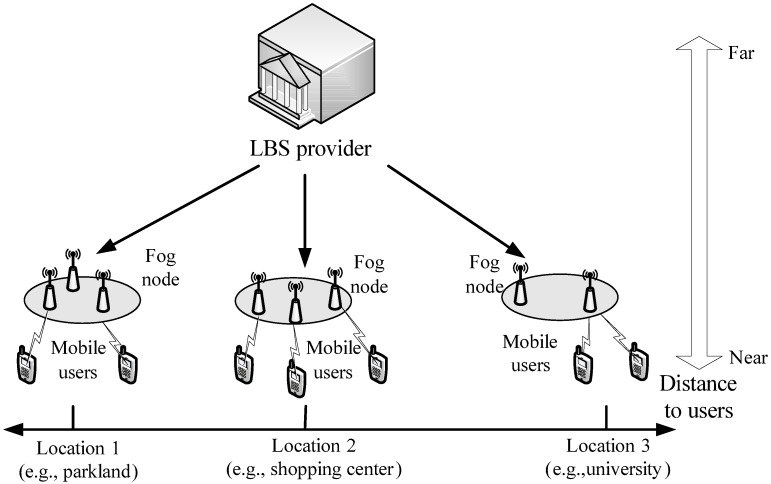
System model under consideration.

We illustrate an overall architecture of fog computing-enhanced LBS query in Figure 1, which includes a set of mobile users, a set of fog nodes $\left\{id_{1},id_{2},\ldots ,id_{n}\right\}$ and an LBS provider.

- LBS provider: the LBS provider acts as a profit company, providing the location-based services for the registered mobile users. With the advantages of fog computing, the LBS provider prefers to outsource its LBS data containing service content and the corresponding geographic location to appropriate fog nodes based on the geographic location distance, which can provide the low-latency LBS for mobile users.
- Fog nodes $\left\{id_{1},id_{2},\ldots ,id_{n}\right\}$: with the pay-as-you-use way, each fog node $id_{j}$ stores the LBS data from the LBS provider and provides the fine-grained query services for mobile users.
- Mobile users: a mobile user who acts as a registered member of the LBS provider, sends a query that contains the searching content, current location information and searching spatial range, to a local fog node for requesting the nearby POIs satisfying both the given searching content and spatial range.

Similar to the most common assumption in research literature (see [27,28]), fog nodes are considered honest-but-curious, which honestly follow the underlying scheme, but are curious about the privacy of mobile users and the LBS provider. In the LBS applications, the LBS provider or mobile users would not collude with fog nodes to obtain others’ privacy. This is because if the LBS provider agree to collude with a fog node to obtain the content of a user’ request, then this node can obtain some LBS data considered as provider’s privacy from the LBS searching. For example, if a fog node obtain a mobile user’s request (e.g., a hospital within 500 m) from the collusion attack, then it can obtain the real information of the matched LBS data, i.e., it knows that the service content of all matched LBS data is the hospital and the corresponding location is within 500 m of the current location. In other words, once the privacy of mobile users is disclosed, the privacy of the LBS provider will also be disclosed. Therefore, the LBS provider would not collude with fog nodes. Similarly, mobile users would also not collude with fog nodes. Note that, since the privacy preservation is our focus, some active attacks are beyond the scope of this work and will be discussed in the future.

### 2.2. Design Goals

The goals of our proposed FGPQ scheme are described as follows:

- Privacy preservation. As a profit company, the LBS data are considered as the LBS provider’s own asset, which should be protected from disclosing. Therefore, the LBS data should be encrypted before being outsourced to fog nodes. For mobile users, a service query may contain some sensitive information, e.g., hobbies, current location and the location where mobile users will reach, which should also be protected from disclosing. Hence, mobile users should send the encrypted query request to the nearby fog node.
- Fine-grained query result. Besides the searching spatial range, the FGPQ scheme should satisfy the searching content, e.g., a hospital or a restaurant. That is, the query result should satisfy the given searching content and searching spatial range simultaneously.
- Efficiency. Owing to the mobility of mobile users, the low-latency is very critical for the LBS [9]. Hence, computational costs and communication delay should be as less as possible.

## 3. Preliminaries

In this section, we first outline the bilinear pairing [25], which will be used for generating the index of the service content to complete the content matching. Then, we describe the asymmetric scalar-product preserving encryption algorithm (ASPE) [26], which will be applied to achieve the encrypted location matching.

### 3.1. Bilinear Pairing

Given a security parameter $\kappa \in Z^{+}$, a bilinear-parameter generation algorithm G(κ) outputs a tuple $\left(p,g,G,G_{T},e\right)$, where *p* is a κ-bit prime number, G and $G_{T}$ are two multiplicative cyclic groups of order *p*, *g* is a generator of G, and $e:G\times G\to G_{T}$ is a bilinear pairing with the following properties:

- Bilinearity: for all u,v∈G and $a,b\in Z_{p}^{*}$, we have $e\left(u^{a},v^{b}\right)=e{\left(u,v\right)}^{ab}$.
- Non-degeneracy: e(g,g)≠1.

The definitions of bilinear pairing parameter generator and the related complexity assumptions are described below [25,29].

**Definition** **1.** * (Bilinear generator) The bilinear parameter generator Gen(·) is a probabilistic algorithm that takes a security parameter κ as input and outputs a 5-tuple $\left(G,G_{T},p,g,e\right)$.*

**Definition** **2.** (Computational Diffie–Hellman (CDH) problem) Given $(g,g^{a},g^{b})\in G$, for unknown $a,b\in Z_{p}^{*}$, compute $g^{ab}\in G$.

**Definition** **3.** (Discrete logarithm (DL) problem) Given Q∈G, compute $a\in Z_{p}^{*}$ such that $g^{a}=Q$.

### 3.2. The Asymmetric Scalar-Product Preserving Encryption (ASPE)

Wong et al. [26] developed an asymmetric scalar-product preserving encryption (ASPE), which has been widely applied to the outsourced range matching in the ciphertext [30,31]. Hence, this encryption algorithm can also applied to the outsourced spatial range query in the ciphertext.

- Key generation (Gen(d)). Given a security parameter *d*, two d×d invertible matrices $M_{1}$ and $M_{2}$, and a *d*-dimensional binary vector *S* are chosen as the private key, denoted as $sk=(S,M_{1},M_{2})$. Note that the binary vector *S* is a splitting indicator to split the plaintext vector into two random vectors, where S[i] is the *i*-th bit in *S*.
- Tuple encryption function $E_{T}\left(\cdot \right)$. Consider a *d*-dimensional vector *P* in a database. Firstly, split *P* into two *d*-dimensional vectors $\left(P_{a},P_{b}\right)$ based on the splitting indicator *S*. Specifically, if S[i]=0(i=1,2,…,d), $P_{a}\left[i\right]=P_{b}\left[i\right]=P\left[i\right]$; if S[i]=1(i=1,2,…,d), the value of P[i] will be randomly split into $P_{a}\left[i\right]$ and $P_{b}\left[i\right]$ such that $P\left[i\right]=P_{a}\left[i\right]+P_{b}\left[i\right]$. Then, the encrypted value of *P* can be calculated as
  (1) $$E_{T}\left(P,sk\right)=\left(P_{a}M_{1},P_{b}M_{2}\right).$$
- Query encryption function $E_{Q}\left(\cdot \right)$. Consider a *d*-dimensional vector *Q*, split it into two *d*-dimensional vectors $\left(Q_{a},Q_{b}\right)$: if S[i]=0(i=1,2,…,d), the value of Q[i] can be randomly split into $Q_{a}\left[i\right]$ and $Q_{b}\left[i\right]$ with $Q\left[i\right]=Q_{a}\left[i\right]+Q_{b}\left[i\right]$; if S[i]=1(i=1,2,…,d), $Q_{a}\left[i\right]=Q_{b}\left[i\right]=Q\left[i\right]$. The encrypted value of *Q* can be generated as
  (2) $$E_{Q}\left(Q,sk\right)=\left(M_{1}^{-1}Q_{a}^{T},M_{2}^{-1}Q_{b}^{T}\right),$$
  where $Q_{a}^{T}$ and $Q_{b}^{T}$ denote the transpose of $Q_{a}$ and $Q_{b}$, respectively.
- Outsourced scalar-product calculation. With two ciphertexts $E_{T}\left(P,sk\right)$ and $E_{Q}\left(Q,sk\right)$, the outsourced scalar-product can be calculated as
  $$\begin{array}{ccc} E_{T}\left(P,sk\right)\cdot E_{Q}\left(Q,sk\right) & = & \left(P_{a}M_{1},P_{b}M_{2}\right)\cdot \left(M_{1}^{-1}Q_{a}^{T},M_{2}^{-1}Q_{b}^{T}\right)\\ & = & P_{a}M_{1}\cdot M_{1}^{-1}Q_{a}^{T}+P_{b}M_{2}\cdot M_{2}^{-1}Q_{b}^{T}\\ & = & P_{a}\cdot Q_{a}^{T}+P_{b}\cdot Q_{b}^{T}\\ & = & P\cdot Q. \end{array}$$
  The correctness of the scalar-product calculation can be referred to [26].
- Decryption Function D(·). Consider an encrypted value $\left(P_{a}M_{1},P_{b}M_{2}\right)$. Firstly, compute the inverse matrices $M_{1}^{-1}$ and $M_{2}^{-1}$, and then extract two vectors $P_{a}$ and $P_{b}$, i.e., $P_{a}=P_{a}M_{1}\cdot M_{1}^{-1}$, $P_{b}=P_{b}M_{2}\cdot M_{2}^{-1}$. Finally, recover the original *d*-dimensional vector *P* with the splitting indicator *S*:
  $$P\left[i\right]=\left\{\begin{array}{ccc} P_{a}\left[i\right]=P_{b}\left[i\right], & if & S[i]=0,\\ P_{a}\left[i\right]+P_{b}\left[i\right], & if & S[i]=1. \end{array}\right.$$

*The Properties of ASPE Algorithm:* For any encrypted tuple $E_{T}\left(P_{i},sk\right)$ and an encrypted query $E_{Q}\left(Q,sk\right)$, the ASPE algorithm satisfies the following two properties:

- For any $P_{i}$ encrypted by $E_{T}\left(\cdot \right)$ and any query *Q* encrypted by $E_{Q}\left(\cdot \right)$:
  (3) $$P_{i}\cdot Q=E_{T}\left(P_{i},sk\right)\cdot E_{Q}\left(Q,sk\right).$$
- For any $P_{i}$ and $P_{j}$ encrypted by $E_{T}\left(\cdot \right)$:
  (4) $$P_{i}\cdot P_{j}\neq E_{T}\left(P_{i},sk\right)\cdot E_{T}\left(P_{j},sk\right).$$

Note that the actual dimension *d* may be too small to resist the brute-force attack. Therefore, it is better to improve the system security by making *d* larger, which can be achieved by adding *artificial* dimensions to the data vector. In particular, extend a *d*-dimensional vector to a $d^{\prime }$-dimensional ($d\leq d^{\prime }$) by padding artificial data such that the scalar-product over the added data is 0. In addition, $d^{\prime }\geq 80$ is large enough to make the system security. The details can be referred to [26]. Unless other specification, the encryption $E_{T}\left(P,sk\right)$ and $E_{Q}\left(Q,sk\right)$ are denoted by $E_{T}\left(P\right)$ and $E_{Q}\left(Q\right)$ for short in the rest of this paper, respectively.

## 4. Proposed FGPQ Scheme

This section presents a fine-grained and privacy-preserving query scheme for fog computing-enhanced LBS (FGPQ), which mainly consists of two parts: system initialization and privacy-preserving LBS query. In particular, the LBS provider mainly initializes the LBS system in the system initialization part, which includes the generation of public and private keys, and LBS data encryption. The privacy-preserving LBS query completes the LBS query process, which includes query request generation, LBS searching and result retrieval. The details are shown as follows.

### 4.1. System Initialization

The LBS provider initializes the system by following steps:

1. Key generation. The LBS provider generates the public and private keys, which are used to encrypt the LBS data and the query request. Specifically, the LBS provider first generates the bilinear pairing tuple $\left(p,g,G,G_{T},e\right)$ and the ASPE algorithm’s private key $\left(S,M_{1},M_{2}\right)$, respectively. Then, it chooses a large random number $\alpha \in Z_{p}^{*}$ as the master-key, and a cryptographic hash function $h:{\left\{0,1\right\}}^{*}\to Z_{p}^{*}$. In addition, in our proposed scheme, the actual dimension *d* of the ASPE algorithm is 7, which can not ensure the sufficient security; thus, we should extend *d* to $d^{\prime }=80$. To this end, the LBS provider also chooses $\left(d^{\prime }-d\right)$ random numbers $\omega_{d+1},\omega_{d+2},\ldots ,\omega_{d^{\prime }}$ as the private key of the ASPE algorithm. After that, the LBS provider publishes $\left(p,g,G,G_{T},e,h\right)$ as the public key. Once a mobile user is registered in the LBS provider, the LBS provider will assign the private key $\left(\alpha ,S,M_{1}^{-1},M_{2}^{-1},\omega_{d+1},\ldots ,\omega_{d^{\prime }}\right)$ to mobile users through the secure channel.
2. LBS data encryption. As mentioned in [18,19], the LBS provider has all LBS data in the system, which can be denoted as $\left\{\left(m_{1},\left(X_{1},Y_{1}\right)\right),\ldots ,\left(m_{k},\left(X_{k},Y_{k}\right)\right)\right\}$. In particular, $m_{i}$ and $\left(X_{i},Y_{i}\right)$ denote the service content and the corresponding location for *i*-th service, respectively. In order to protect the privacy, the LBS provider encrypts the LBS data and separately sends them to appropriate fog nodes. Concretely, for each service item $\left(m_{i},\left(X_{i},Y_{i}\right)\right)$, where i=1,2,…,k, the LBS provider conducts the following calculations.
  - For the service content $m_{i}$, the LBS provider chooses a random number $r_{i}\in Z_{p}^{*}$, and generates the ciphertext as
    $$E\left(m_{i}\right)=\left(g^{\alpha h\left(m_{i}\right)r_{i}},g^{r_{i}}\right).$$
  - For the location information $\left(X_{i},Y_{i}\right)$, the LBS provider first generates a 7-dimensional vector as $W^{\left(i\right)}=\left(1,1,X_{i}^{2},Y_{i}^{2},-2X_{i},-2Y_{i},-1\right)$, and extends it to a $d^{\prime }$-dimensional vector $\hat{W^{\left(i\right)}},$ where the first seven dimensions are copied from $W^{\left(i\right)}$, and for j=8 to $d^{\prime }-1$, set $\hat{W^{\left(i\right)}}\left[j\right]=r_{j}$ ($r_{j}$ is a random number), and $\hat{W^{\left(i\right)}}\left[d^{\prime }\right]=\frac{-\sum_{j=8}^{d^{\prime }-1}\omega_{j}r_{j}}{\omega_{d^{\prime }}}$, i.e., $\hat{W^{\left(i\right)}}=\left(1,1,X_{i}^{2},Y_{i}^{2},-2X_{i},-2Y_{i},-1,r_{8},r_{9},\ldots ,r_{d^{\prime }-1},\frac{-\sum_{j=8}^{d^{\prime }-1}\omega_{j}r_{j}}{\omega_{d^{\prime }}}\right)$. Then, the LBS provider encrypts $\hat{W^{\left(i\right)}}$ with the private key $\left(S,M_{1},M_{2}\right)$ by means of the tuple encryption function $E_{T}\left(\cdot \right)$ (the Equation (1)), that is,
    $$E_{T}\left(\hat{W^{\left(i\right)}}\right)=\left(\hat{W_{a}^{\left(i\right)}}\cdot M_{1},\;\hat{W_{b}^{\left(i\right)}}\cdot M_{2}\right),$$
    where $\hat{W_{a}^{\left(i\right)}}$ and $\hat{W_{b}^{\left(i\right)}}$ are two split vectors of $\hat{W^{\left(i\right)}}$.

Finally, based on the geographic location distance, the LBS provider outsources all *k* encrypted LBS data $\left(E\left(m_{i}\right),E_{T}\left(\hat{W^{\left(i\right)}}\right)\right)$, where i=1,2,…,k, to appropriate fog nodes.

### 4.2. Privacy-Preserving LBS Query

This section achieves the privacy-preserving LBS query by means of the bilinear pairing and the ASPE algorithm, which includes LBS query request generation, LBS searching and request result decryption. Before that, we first define a range query used for location range matching.

**Definition** **4.** Given the location coordinate $\left(x_{*},y_{*}\right)$ and the searching spatial range T, a location coordinate $\left(x_{i},y_{i}\right)$ is within the searching spatial range if and only if

(5) $$R=\sqrt{{\left(x_{*}-x_{i}\right)}^{2}+{\left(y_{*}-y_{i}\right)}^{2}}\leq T,$$

*where R is the Euclidean distance used to compute the distance between two coordinates [32].*

#### 4.2.1. LBS Query Request Generation

When a mobile user wants to search for a POI within a certain range, e.g., a hospital within 1000 m of the current location, he should send the query request that contains the searching content $m_{*}$ (a hospital), the current location $\left(X_{*},Y_{*}\right)$ and the searching spatial range $T_{*}$ (within 1000 m), to a local fog node (denoted as $id_{j}$). In order to ensure the privacy, he encrypts the request information $\left(m_{*},\left(X_{*},Y_{*}\right),T_{*}\right)$ firstly, and then sends the encrypted query request to the fog node $id_{j}$. The details are described as follows.

1. For the searching content $m_{*}$, the user chooses a random number $r_{*}\in Z_{p}^{*}$, and generates the ciphertext as
  $$E\left(m_{*}\right)=\left(g^{\alpha h\left(m_{*}\right)r_{*}},g^{r_{*}}\right).$$
2. For the location information $\left(X_{*},Y_{*}\right)$ and the searching spatial range $T_{*}$, the user first generates a 7-dimensional vector as $L=(X_{*}^{2},Y_{*}^{2},1,1,X_{*},Y_{*},T_{*}^{2})$, and extends it to a $d^{\prime }$-dimensional vector $\hat{L}$ where the first seven dimensions are copied from *L*, and for i=8 to $d^{\prime }$, set $\hat{L}\left[i\right]=\omega_{i}$, i.e., $\hat{L}=\left(X_{*}^{2},Y_{*}^{2},1,1,X_{*},Y_{*},T_{*}^{2},\omega_{8},\omega_{9},\ldots ,\omega_{d^{\prime }}\right)$. Then, the user chooses a random large positive number β to confuse $\hat{L}$, and computes ${\hat{L}}^{\prime }=\beta \cdot \hat{L}$. After that, the user encrypts ${\hat{L}}^{\prime }$ with the private key $\left(S,M_{1}^{-1},M_{2}^{-1}\right)$ by means of the query encryption function $E_{Q}\left(\cdot \right)$ (the Equation (2)) as
  $$E_{Q}\left({\hat{L}}^{\prime }\right)=\left(M_{1}^{-1}\cdot {\hat{L}}_{a}^{\prime },\;M_{2}^{-1}\cdot {\hat{L}}_{b}^{\prime }\right),$$
  where ${\hat{L}}_{a}^{\prime }$ and ${\hat{L}}_{b}^{\prime }$ are two column vectors split from ${\hat{L}}^{\prime }$.

Finally, the user sends the encrypted query request $\left\{E\left(m_{*}\right),E_{Q}\left({\hat{L}}^{\prime }\right)\right\}$ to the fog node $id_{j}$.

#### 4.2.2. LBS Searching

After receiving the encrypted query request $\left\{E\left(m_{*}\right),E_{Q}\left({\hat{L}}^{\prime }\right)\right\}$, the fog node $id_{j}$ completes the LBS searching to find the most matched LBS item, which not only meets both the searching content and spatial range, but also has the shortest distance from the user’s location. Specifically, suppose the fog node $id_{j}$ receives $k_{j}$ encrypted LBS items from the LBS provider, denoted as $\left\{\left(E\left(m_{1}\right),E_{T}\left(\hat{W^{\left(1\right)}}\right)\right),\left(E\left(m_{2}\right),E_{T}\left(\hat{W^{\left(2\right)}}\right)\right),\ldots ,\left(E\left(m_{k_{j}}\right),E_{T}\left(\hat{W^{\left(k_{j}\right)}}\right)\right)\right\}$. For each $\left(E\left(m_{i}\right),E_{T}\left(\hat{W^{\left(i\right)}}\right)\right)$, where $i=1,2,\ldots ,k_{j}$, the fog node $id_{j}$ executes the following operations.

1. For $E_{T}\left(\hat{W^{\left(i\right)}}\right)$, the fog node $id_{j}$ first computes
  (6) $$\begin{array}{ccc} R_{i} & = & E_{T}\left(\hat{W^{\left(i\right)}}\right)\cdot E_{Q}\left({\hat{L}}^{\prime }\right)=\left(\hat{W_{a}^{\left(i\right)}}M_{1},\;\hat{W_{b}^{\left(i\right)}}M_{2}\right)\cdot \left(M_{1}^{-1}{\hat{L}}_{a}^{\prime },\;M_{2}^{-1}{\hat{L}}_{b}^{\prime }\right)\\ & = & \hat{W_{a}^{\left(i\right)}}M_{1}\cdot M_{1}^{-1}{\hat{L}}_{a}^{\prime }+\hat{W_{b}^{\left(i\right)}}M_{2}\cdot M_{2}^{-1}{\hat{L}}_{b}^{\prime }\\ & = & \hat{W_{a}^{\left(i\right)}}{\hat{L}}_{a}^{\prime }+\hat{W_{b}^{\left(i\right)}}{\hat{L}}_{b}^{\prime }\\ & = & \hat{W^{\left(i\right)}}\cdot {\hat{L}}^{\prime }\\ & = & \beta ({\left(X_{*}-X_{i}\right)}^{2}+{\left(Y_{*}-Y_{i}\right)}^{2}-T_{*}^{2}+\omega_{8}r_{8}+\cdots +\omega_{d^{\prime }-1}r_{d^{\prime }-1}-\sum_{j=8}^{d^{\prime }-1}\omega_{j}r_{j})\\ & = & \beta ({\left(X_{*}-X_{i}\right)}^{2}+{\left(Y_{*}-Y_{i}\right)}^{2}-T_{*}^{2}). \end{array}$$
  Then, it checks whether $R_{i}\leq 0$ holds. If not, it means that the LBS item $\left(E\left(m_{i}\right),E_{T}\left(\hat{W^{\left(i\right)}}\right)\right)$ does not satisfy the searching spatial range.
2. If $R_{i}\leq 0$ holds, similarly to [33], the fog node $id_{j}$ checks whether
  (7) $$e\left(g^{\alpha h\left(m_{i}\right)r_{i}},g^{r_{*}}\right)=e\left(g^{\alpha h\left(m_{*}\right)r_{*}},g^{r_{i}}\right)$$
  holds. If not, it means that the service content $m_{i}$ does not satisfy the given searching content $m_{*}$. Otherwise, the LBS item $\left(m_{i},\left(X_{i},Y_{i}\right)\right)$ satisfy the searching condition given by mobile users.

Eventually, the fog node $id_{j}$ sends the matched result $E_{T}\left(\hat{W^{\left(i\right)}}\right)$ to the user. Note that if the service $m_{*}$ has multi-matched locations, the fog node $id_{j}$ always returns the nearest location from the user (the location with the smallest $R_{i}$).

#### 4.2.3. Request Result Decryption

After receiving the result, e.g., $E_{T}\left(\hat{W^{\left(i\right)}}\right)=\left(\hat{W_{a}^{\left(i\right)}}M_{1},\;\hat{W_{b}^{\left(i\right)}}M_{2}\right)$, the user recovers the vector $\hat{W^{\left(i\right)}}$ by means of the decryption function *D*, and then extracts the first seven dimensions in it, i.e., $W^{\left(i\right)}=\left(1,1,X_{i}^{2},Y_{i}^{2},-2X_{i},-2Y_{i},-1\right)$. According to $W^{\left(i\right)}$, the user can obtain the matched location $\left(X_{i},Y_{i}\right)$ about the searching content $m_{*}$.

## 5. Privacy Analysis

Following our design goals, we discuss how the proposed FGPQ scheme achieves the privacy preservation of the LBS provider and mobile users.

The privacy preservation of mobile users: When a mobile user wants to search the POI satisfying the special spatial range and searching content, he or she sends an encrypted request $\left(E\left(m_{*}\right),E_{Q}\left({\hat{L}}^{\prime }\right)\right)$ to the fog node $id_{j}$. In particular, the ciphertext $E(m_{*})$ is computed as $E\left(m_{*}\right)=\left(g^{\alpha h\left(m_{*}\right)r_{*}},g^{r_{*}}\right)$. Hence, based on hard problems of the CDH and DL in bilinear pairing (Definitions 2 and 3), the fog node $id_{j}$ can not obtain any plaintext information from $E(m_{*})$ without knowing $r_{*}$ and α. As $E_{Q}\left({\hat{L}}^{\prime }\right)$ is a ciphertext of the ASPE algorithm, the privacy-preserving totally depends on the security of the ASPE algorithm. As analysed in [26], once the vector dimension *d* satisfies d≥80, it is generally very difficult for an attacker to successfully attack the ASPE algorithm. Therefore, under the parameter setting in Section 4.1, the location can be protected from disclosing. In other words, fog nodes can not obtain any plaintext information from the request $\left(E\left(m_{*}\right),E_{Q}\left({\hat{L}}^{\prime }\right)\right)$. In addition, even if the value of scalar-product (see Formula (6)) has been obtained, the fog node $id_{j}$ cannot get the location, since a large random number β has been applied to confuse the real scalar-product value. Note that fog nodes do not collude with the LBS provider in this paper, so they will not directly forward users’ encrypted requests to the LBS provider, thereby, the LBS provider will not obtain users’ privacy. Consequently, the privacy-preserving of mobile users can be achieved.

The privacy preservation of the LBS provider: Under the aforementioned scheme, the LBS provider would encrypt all LBS data before outsourcing. Thus, fog nodes can only obtain the encrypted LBS items. Similarly, the fog node $id_{j}$ can not obtain any plaintext information from the stored content information $E\left(m_{i}\right)\;\left(i=1,2,\ldots ,k_{j}\right)$ based on the CDH and DL problems. In addition, according to properties of the ASPE algorithm (Formulas (3) and (4)), even though the fog node $id_{j}$ manages $k_{j}$ encrypted LBS items, it can not obtain any plaintext information about locations. Conceivably, the privacy preservation of the LBS provider can be achieved.

## 6. Performance Analysis

In this section, we evaluate our proposed FGPQ scheme in terms of the communication overhead, computational costs and latency, respectively. Moreover, we give a comparison with the EPLQ scheme [18] and EPQ scheme [19].

### 6.1. Computational Costs

In our FGPQ scheme, the LBS provider needs to encrypt *k* LBS data, where each service content $m_{i}$ is encrypted by the bilinear pairing technology, i.e., $e(g^{\alpha h\left(m_{i}\right)r_{i}},g^{r_{i}})$, and the correponding location $\left(X_{i},Y_{i}\right)$ is encrypted by the tuple encryption function $E_{T}$ of the ASPE algorithm. According to the empirical evaluation in [26,30], the computational costs for the ASPE algorithm are microseconds, which are considered negligible compared to the exponentiation and pairing operations. Hence, the computational costs of the LBS provider are 2k exponentiations in G. When a mobile user wants to request the LBS searching, he or she encrypts the query request $\left(m_{*},\left(X_{*},Y_{*}\right),T_{*}\right)$, which costs two exponentiations in G. After receiving the encrypted request, for each encrypted LBS item $\left(E\left(m_{i}\right),E_{T}\left(\hat{W^{\left(i\right)}}\right)\right)$, where $i=1,2,\ldots ,k_{j}$, the fog node $id_{j}$ first computes the Formula (6) to select the LBS items meeting the spatial range. After retrieving *f* LBS items matched the spatial range, it verifies the Equation (7), where each verification requires two pairing operations. Since the computational costs of the ASPE algorithm are negligible, the computational costs of the fog node $id_{j}$ are 2f pairing operations. Finally, the total computational costs for the LBS provider, a mobile user and a fog node $id_{j}$ will be 2k exponentiations in G, two exponentiations in G and 2f pairing operations, respectively, in the proposed FGPQ scheme.

In the EPLQ scheme [18], to generate a query, a mobile user needs to encode the location and the searching spatial range into two *N*-dimensional vectors, and then encrypt these two vectors as $K_{i}=\left(g^{u_{i,1}^{{}^{\prime \prime }}},g^{u_{i,2}^{{}^{\prime \prime }}},\ldots ,g^{u_{i,N}^{{}^{\prime \prime }}},e{\left(g,g\right)}^{h_{i}}\right)$, where $\left(u_{i,1}^{{}^{\prime \prime }},u_{i,2}^{{}^{\prime \prime }},\ldots ,u_{i,N}^{{}^{\prime \prime }}\right)$ is the encoded vector. Hence, the corresponding computational costs are about 2N exponentiations in G. In order to ensure the privacy, the LBS provider also needs to encode the location of each LBS into a *N*-dimensional vectors, and then encrypt it as $C_{j}=\left(g^{v_{j,1}^{{}^{\prime \prime }}},g^{v_{j,2}^{{}^{\prime \prime }}},\ldots ,g^{v_{j,N}^{{}^{\prime \prime }}},e{\left(g,g\right)}^{s_{j}}\right)$, which requires *N* exponentiations in G and one exponentiation in $G_{T}$. For all *k* LBS items, it totally spends N*k exponentiations in G and *k* exponentiations in $G_{T}$ to complete the encryption. In addition, to assist the cloud in checking the spatial range, the LBS provider still needs to compute $\tau_{2}-\tau_{1}$ values $\Omega_{i}=Hash\left(e{\left(g,g\right)}^{{\left(\alpha \times perm\left(i\right)+\beta \right)}^{d}}\right)$, where $i\in [\tau_{1},\tau_{2}]$. That is, the computational costs are about $\left(\tau_{2}-\tau_{1}\right)$ exponentiations in $G_{T}$. Hence, the total computational costs for the LBS provider are about N*k exponentiations in G and $k+(\tau_{2}-\tau_{1})$ exponentiations in $G_{T}$. To determine whether the encrypted LBS items match the given query or not, it requires the cloud to compute $check(K_{i},C_{j})$, which costs *N* pairing operations and *N* multiplications in $G_{T}$. Note that searching $\hat{ss}$-tree to find the matched LBS items requires traversing through about logk+f tree nodes with *k* LBS items and *f* items matched the spatial range. Therefore, the total computational costs for the cloud are about (logk+f)N pairing operations and (logk+f)N multiplications in $G_{T}$.

In the EPQ scheme [19], the LBS provider outsources all encrypted LBS items to the cloud, where each location index is encrypted as $l_{s1}=e{\left(g,g\right)}^{q_{1}{x_{s_{0}}}^{2}}$, $l_{s2}=e{\left(g,g\right)}^{q_{1}{y_{s_{0}}}^{2}}$, $l_{s3}=g^{x_{s_{0}}}\cdot h^{r_{s_{1}}}$ and $l_{s4}=g^{x_{s_{0}}}\cdot h^{r_{s_{2}}}$. The corresponding computational costs for all *k* LBS items are about 4k exponentiations in G, 2k exponentiations in $G_{T}$ and 2k multiplications in G. To request a LBS query, a mobile user generates an encrypted query $\left(rq_{1},rq_{2},rq_{3},rq_{4}\right)=\left(e{\left(g,g\right)}^{q_{1}\left({x_{s_{0}}}^{2}-d^{2}\right)},e{\left(g,g\right)}^{q_{1}{y_{0}}^{2}},g^{q_{1}\cdot 2x_{0}},g^{q_{1}\cdot 2y_{0}}\right)$, which requires two exponentiations in G and two exponentiations in $G_{T}$. After receiving the query from the user, the cloud computes the search criteria $T_{s}$ for each LBS item stored in it, which requires 2k pairing operations and 5k multiplications in $G_{T}$.

We present the computational costs comparison of our FGPQ scheme, the EPLQ scheme [18] and the EPQ scheme [19] in Table 1, where *k* and *f* denote the number of LBS data items and the matched LBS data items, respectively. Obviously, *k* and *f* satisfy f≤k, and the codeword length *N* satisfies N≥7 [18]. Note that $C_{e}$, $C_{et}$, $C_{p}$, $C_{mt}$ and $C_{m}$ denote the computational costs of an exponentiation in G, an exponentiation in $G_{T}$, a pairing operation, a multiplication in $G_{T}$ and a multiplication in G, respectively. From the table, it is obvious that the FGPQ scheme largely reduces the computational costs compared to the EPLQ scheme [18] and the EPQ scheme [19]. Moreover, for mobile users, the efficiency of our FGPQ scheme is better than the others. Thus, as declared in [18,19], it is convincing that our FGPQ scheme is more suitable for energy-poor mobile devices.

### 6.2. Communication Overhead

In our FGPQ scheme, the communication overhead can be divided into two parts: LBS provider-to-fog and user-to-fog. We first discuss the LBS provider-to-fog communication, where the LBS provider delivers all encrypted LBS items to each fog node $id_{j}\;\left(j=1,2,\ldots ,n\right)$. The encrypted LBS item is in the form of ${\left\{E\left(m_{i}\right),E_{T}\left(\hat{W^{\left(i\right)}}\right)\right\}}_{i=1,2,\ldots ,k_{j}}$ for the fog node $id_{j}$, and its size should be $\left(1024+\right|E_{T}\left(\hat{W^{\left(i\right)}}\right)\left|\right)k_{j}$ bits if we choose 512-bit G. As mentioned in [30], each dimension in the ASPE algorithm is a float number (the size of each float number is 32 bits). Thus, the total size of $E_{T}\left(\hat{W^{\left(i\right)}}\right)=\left(\hat{W_{a}^{\left(i\right)}}M_{1},\hat{W_{b}^{\left(i\right)}}M_{2}\right)$ is 5120 bits if the dimension is 80 (see Section 4.1). Accordingly, the communication overheads for LBS provider-to-fog communication are $\sum_{j=1}^{n}\left(6144\cdot k_{j}\right)$ bits. As mentioned in Section 4.2.2, since all *k* encrypted LBS items are distributed to *n* fog nodes, we can obtain $\sum_{j=1}^{n}k_{j}=k$, where $k_{j}$ denotes the number of fog node $id_{j}$’s LBS items received from the LBS provider. That is, the value $\sum_{j=1}^{n}\left(6144\cdot k_{j}\right)$ can be implicitly simplified as (6144·k). Next, we consider the user-to-fog communication. Firstly, a mobile user sends an encrypted request $\left\{E\left(m_{*}\right),E_{Q}\left({\hat{L}}^{\prime }\right)\right\}$ to a fog node, which requires 6144 bits to transmit. Then, this fog node performs the LBS searching and returns the most matched encrypted location $E_{T}\left(\hat{W^{\left(i\right)}}\right)$ to this user, which costs 5120 bits. Therefore, the total communication overheads for user-to-fog communication are about 11,264 bits.

In the EPLQ scheme [18], the LBS provider outsources all encrypted LBS items to the cloud. The communication overheads are $\left(N+1\right)k\cdot 512+k\cdot 128+128\cdot \left(\tau_{2}-\tau_{1}\right)$ bits if the bit length of *p*, standard symmetric encryption (e.g., AES) and the hash value hash() satisfy |p|=512 bit, |AES|=128 bit and |hash()|=128 bit, respectively. To achieve the LBS searching, a mobile user sends two encrypted tokens to the cloud, which requires 2(N+1)·512 bits. After receiving the LBS searching request, the cloud performs LBS checking to find the LBS items matched the given spatial range. After that, the cloud returns the matched LBS data items to this user. If the number of matched LBS items is *f*, then the communication overheads are about f·128 bits, where each ciphertext of the symmetric encryption is 128 bits. Therefore, the LBS provider-to-cloud communication and the user-to-cloud communication require $\left(512\cdot \left(N+1\right)k+128\cdot k+128\times 10^{8}\right)$ bits and (1024·(N+1)+128·f) bits, respectively.

In the EPQ scheme [19], in order to enjoy the location-based services, a mobile user sends an encrypted LBS query request to the cloud. The encrypted LBS query request is in the form of $\left(ID_{LBS}||E_{LQR}||U_{i}||TS||Sig_{i}\right)$, and its size is about 5280 bits if we set $|ID_{LBS}\left|\right|U_{i}\left|\right|TS|=160$ bits. To provide the better quality of services, the LBS provider outsources all encrypted LBS data to the cloud. Since each outsourced LBS item is in the form of $\left(ID_{LBS}||T_{j}||l_{s_{1}}||l_{s_{2}}||l_{s_{3}}||l_{s_{4}}||E_{s}\right)$, the communication overheads for all *k* LBS items are about 4384·k bits. In addition, in order to help the cloud to complete the query in the ciphertexts, the LBS provider also sends the evaluation data set $EDS=\{ED_{0},ED_{1},\ldots ,ED_{i},\ldots ,ED_{10,000,000,000}\}$ to the cloud, where $ED_{i}=H\left(PB^{i}\right)$. If the bit length of hash H() is set as |H()|=128 bit, then the size of the set EDS is about $128\times 10^{10}$ bits. Hence, the communication overheads for the LBS provider are about $\left(4384\cdot k+128\times 10^{10}\right)$ bits. After finding *f* matched LBS items, the cloud returns the query result $\left(E_{rq_{1}}\left(TRL\right)||ID_{CS}||TS||Sig_{CS}\right)$ to the user, which requires (128·f+1184) bits to transmit. Hence, the total communication overheads between a mobile user and the cloud are about (128·f+6464) bits.

We present the communication overhead comparison of our FGPQ scheme, the EPLQ scheme [18] and the EPQ scheme [19] in Table 2, where “LBS provider-to-cloud (fog nodes)” and “ User-to-cloud (a fog node) ” denote the communication between the LBS provider and the cloud (or fog nodes) and the communication between a mobile user and the cloud (or a fog node), respectively. As described in the EPLQ scheme [18], the codeword length *N* satisfies N≥7, since the original dimension of the location vector is 7. Therefore, from Table 2, we can know that the communication overheads between the LBS provider and the cloud (or fog nodes) in these three schemes are about the same. However, in the user-to-cloud (a fog node) communication, the communication overhead of our FGPQ scheme is much less than that of other two schemes, as the number of LBS items matched the given spatial range is usually more than a few dozen, i.e., *f* is more than a few dozen, and *f* is likely to increase with *k* or the searching spatial range increasing.

### 6.3. Latency

In order to demonstrate the advantage of fog computing, this section discusses the latency of LBS searching, and gives a comparison with cloud computing-based schemes, e.g., the schemes proposed in [18,19]. In general, the service latency is a time interval from a user sending a request to receiving the feedback, which contains the online computing delay and communication delay. In our FGPQ scheme, since fog nodes are local, the communication delay between mobile users and fog nodes can be neglected, and thus the latency absolutely depends on the online computing time in fog nodes. As shown in Table 1, the online computing time of the fog node $id_{j}$ is $2f\cdot C_{p}$ seconds, that is, the latency of our FGPQ scheme is $2f\cdot C_{p}$ seconds. Alternatively, if the cloud computing system is adopted, the latency depends on the online computing time in the cloud and the communication delay between users and the cloud. Specifically, in the EPLQ scheme [18], the online computing time in the cloud is $\left(\log k+f\right)\cdot N\cdot \left(C_{p}+C_{mt}\right)$ seconds (See Table 1). In addition, based on Table 2, the communication overheads between each user and a fog node are 128·f+1024(N+1) bits. Therefore, if *l* mobile users send requests to the cloud at the same time, the total network traffic is about (128·f+1024(N+1))·l bits. As analysed in [34], the communication delay for uploading 10 MB data takes about 28 s, thereby, the communication delay of the EPLQ scheme [18] is about (128·f+1024(N+1))·l÷8388608 s (1 MB =8,388,608 bits). Finally, the latency of the EPLQ scheme [18] is about $\left(\left(\log k+f\right)\cdot N\cdot \left(C_{p}+C_{mt}\right)+\left(128\cdot f+1024\left(N+1\right)\right)\cdot l÷8388608\right)$ s. Similarity, based on Table 1 and Table 2, the latency of the EPQ scheme [19] is about $\left(2k\cdot C_{p}+5k\cdot C_{mt}+\left(128f+6464\right)l÷8388608\right)$ s.

**Figure 2:**
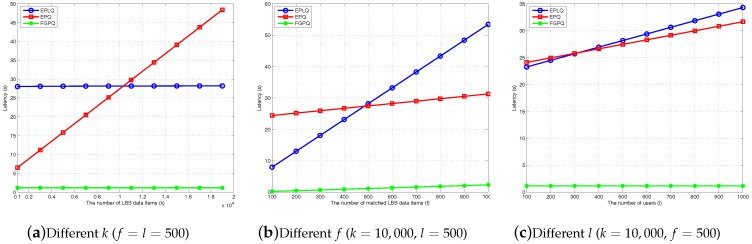
The comparison of latency for location-based service searching.

Furthermore, we conduct the experiments with the pairing-based cryptograph library (PBC) [35] and the GNU multiple precision arithmetic library (GMP) [36] running on a 2.6 GHz-processor computer to study the latency. To this end, we set the security parameter |κ|=512 bits. Furthermore, similar to [18], we set N=37. With the exact operation costs, we depict the variation of computational costs in terms of the number of LBS items *k*, the number of matched LBS items and the number of mobile uses *l* who request LBS searching at the same time, in Figure 2. From the figure, it is obvious that our FGPQ scheme largely reduces the latency compared to the EPLQ scheme [18] and EPQ scheme [19]. In particular, in Figure 2a, our FGPQ scheme remains unchanged when *k* increases. This is because our FGPQ scheme takes advantage of a very efficient ASPE algorithm, and uses this algorithm to originally select *f* encrypted LBS items matched the spatial range. In addition, in practice, the number of LBS data stored in each fog node is far less than the total LBS data (i.e., $k_{j}\ll k$). Therefore, the latency of our FGPQ scheme only depends on the time verifying whether *f* LBS data satisfy the searching content, i.e., 2f pairing operations. In Figure 2c, our FGPQ scheme still remains unchanged when *l* increases. The main reason is that the communication delay between mobile users and local fog nodes can be neglected, and the computing operations for *l* users can be performed in parallel. According to Figure 2b, the latency of these three schemes increase with *f* increasing, but our scheme increase significantly slower.

From the above analyses, our FGPQ scheme is indeed efficient in terms of computational and communication overheads, and also low-latency in fog computing-enhanced system, which is suitable for the real-time LBS searching for mobile users.

## 7. Conclusions

Fog computing-enhanced location-based services have been widely developed in the Internet of Things. In this paper, we have proposed a fine-grained and privacy-preserving query scheme in fog computing-enhanced location-based services. In our proposed scheme, mobile users can obtain the fine-grained searching result satisfying not only the given spatial range but also the searching content. Detailed privacy analysis shows that our proposed scheme indeed achieves the privacy preservation for the LBS provider and mobile users. In addition, extensive performance analyses and experiments demonstrate that our proposed scheme can significantly reduce computational and communication overheads and ensure low-latency, which outperforms existing state-of-the art schemes. Hence, our proposed scheme is more suitable for real-time LBS searching. In future work, we will consider a stronger adversarial model and design new solutions under the new model. In order to achieve more fine-grained LBS queries, we will consider the extensibility, such as searching the closest restaurant that opens now and satisfies the five-star service score or has good sales volume, which makes the LBS query be more practical.

## Figures and Tables

**Table 1:** Comparison of computational costs.

	Mobile User	LBS Provider	Cloud/Fog Node
**EPLQ** [18]	$2N\cdot C_{e}$	$N\cdot k\cdot C_{e}+\left(k+\tau_{2}-\tau_{1}\right)\cdot C_{et}$	$\left(\log k+f\right)N\cdot \left(C_{p}+C_{mt}\right)$
**EPQ** [19]	$2\cdot (C_{e}+C_{et})$	$4k\cdot C_{e}+2k\cdot \left(C_{et}+C_{m}\right)$	$2k\cdot C_{p}+5k\cdot C_{mt}$
**FGPQ**	$2\cdot C_{e}$	$2k\cdot C_{e}$	$2f\cdot C_{p}$

**Table 2:** Comparison of communication overhead (Bits).

	LBS Provider-to-Cloud (Fog Nodes)	User-to-Cloud (A Fog Node)
**EPLQ** [18]	$512\cdot \left(N+1\right)k+128\cdot k+128\cdot \left(\tau_{2}-\tau_{1}\right)$	1024·(N+1)+128·f
**EPQ** [19]	$4384\cdot k+128\times 10^{10}$	128·f+6464
**FGPQ**	6144·k	11264
